# Founder Mutation in N Terminus of Cardiac Troponin I Causes Malignant Hypertrophic Cardiomyopathy

**DOI:** 10.1161/CIRCGEN.120.002991

**Published:** 2020-09-04

**Authors:** Akl C. Fahed, Georges Nemer, Fadi F. Bitar, Samir Arnaout, Antoine B. Abchee, Manal Batrawi, Athar Khalil, Ossama K. Abou Hassan, Steven R. DePalma, Barbara McDonough, Mariam T. Arabi, James S. Ware, Jonathan G. Seidman, Christine E. Seidman

**Affiliations:** 1 Division of Cardiology, Department of Medicine, Center of Genomic Medicine, Massachusetts General Hospital (A.C.F.), Harvard Medical School, Boston.; 2 Department of Genetics (S.R.D., B.M., J.G.S., C.E.S.), Harvard Medical School, Boston.; 3 Cardiovascular Disease Initiative, Broad Institute of MIT and Harvard, Cambridge, MA (A.C.F.).; 4 Department of Biochemistry and Molecular Genetics, American University of Beirut, Lebanon (G.N., F.F.B., M.B., A.K., O.A.-H.).; 5 College of Health and Life Sciences, Hamad Bin Khalifa University, Education City, Doha, Qatar (G.N.).; 6 Department of Pediatrics (F.F.B., M.T.A.), American University of Beirut Medical Center, Lebanon.; 7 Cardiology Division (S.A., A.B.A., O.A.-H.), American University of Beirut Medical Center, Lebanon.; 8 National Heart and Lung Institute, Imperial College London, Royal Brompton Hospital (J.S.W.).; 9 Medical Research College London Institute of Medical Sciences, United Kingdom (J.S.W.).; 10 Division of Cardiology and Howard Hughes Medical Institute, Brigham and Women’s Hospital, Boston, MA (C.E.S.).

**Keywords:** cardiomyopathy, hypertrophic, death, sudden, cardiac, disease, mutation, risk

## Abstract

Cardiac troponin I (*TNNI3*) gene mutations account for 3% of hypertrophic cardiomyopathy and carriers have a heterogeneous phenotype, with increased risk of sudden cardiac death (SCD). Only one mutation (p.Arg21Cys) has been reported in the N terminus of the protein. In model organisms, it impairs PKA (protein kinase A) phosphorylation, increases calcium sensitivity, and causes diastolic dysfunction. The phenotype of this unique mutation in patients with hypertrophic cardiomyopathy remains unknown.

**Methods:**

We sequenced 29 families with hypertrophic cardiomyopathy enriched for pediatric-onset disease and identified 5 families with the *TNNI3* p.Arg21Cys mutation. Using cascade screening, we studied the clinical phenotype of 57 individuals from the 5 families with *TNNI3* p.Arg21Cys-related cardiomyopathy. We performed survival analysis investigating the age at first SCD in carriers of the mutation.

**Results:**

All 5 families with *TNNI3* p.Arg21Cys were from South Lebanon. *TNNI3* p.Arg21Cys-related cardiomyopathy manifested a malignant phenotype—SCD occurred in 30 (53%) of 57 affected individuals at a median age of 22.5 years. In select carriers without left ventricular hypertrophy on echocardiogram, SCD occurred, myocyte disarray was found on autopsy heart, and tissue Doppler and cardiac magnetic resonance imaging identified subclinical disease features such as diastolic dysfunction and late gadolinium enhancement.

**Conclusions:**

The *TNNI3* p.Arg21Cys mutation has a founder effect in South Lebanon and causes malignant hypertrophic cardiomyopathy with early SCD even in the absence of hypertrophy. Genetic diagnosis with this mutation may be sufficient for risk stratification for SCD.

## Abstract 2

Supplemental Digital Content is available in the text.

Hypertrophic cardiomyopathy (HCM) is a disease of cardiac muscle caused by sarcomere gene mutations and is associated with increased risk of sudden cardiac death (SCD).^1,2^ Among patients 10 to 45 years of age, SCD occurs at an annual incidence of <1 in 1000, with the majority occurring in previously undiagnosed individuals.^3^ Of all SCD cases in people aged 5 to 34 years, 14% are due to HCM.^4^ The weak genotype-phenotype correlations and wide phenotypic variability of the disease within and between families limit the ability of using genetics to predict who will experience SCD.^5,6^ For example, while one HCM patient could have unexplained asymmetrical left ventricular hypertrophy at a young age and subsequently experience an SCD, many others have subclinical disease and remain undiagnosed unless detected by genetic screening, often performed after a family member is diagnosed.

An understanding of the phenotype driven by a specific gene mutation with known molecular mechanism could provide an opportunity for more personalized treatment of HCM. If a molecular genotype predicts substantial risk of SCD, then carriers can place an implantable cardioverter-defibrillator—an effective strategy for prevention of SCD in patients with HCM.^7^ Currently, implanting a defibrillator is based on clinical criteria such as the presence of ventricular arrhythmias, syncope or prior cardiac arrest, family history of a close relative with SCD, and massive myocardial thickness.^8^ With few exceptions, using genetic mutations to inform risk stratification for SCD in patients with HCM is limited for 3 reasons. First, most mutations are private precluding the availability of large cohorts with a single mutation. Second, mutations in the same gene can have differential effects on the protein structure and subsequently the phenotype. Third, clinical phenotypes such as cardiac hypertrophy are the end result of different molecular pathways.

Mutations in the gene encoding the cardiac troponin I (*TNNI3*) account to around 3% of HCM and also have a heterogeneous phenotype.^6^ More than 55 mutations in *TNNI3* have been reported to cause cardiomyopathy, mostly hypertrophic but also a minority that can cause a dilated or restrictive cardiomyopathy.^9,10^ Troponin is a protein complex made of troponin I, troponin C, and troponin T and is located within the thin filament of the sarcomere where it is responsible for binding calcium and switching contraction. Upon calcium binding, troponin undergoes a series of conformational changes allowing the release of troponin I inhibition from actin and resulting in actin-myosin binding and force generation. The cardiac troponin I is different from skeletal troponin I in that it has an additional 32 amino-acid sequence on its N-terminal domain. Only 1 HCM-causing mutation, NM_000363.5:c.61C>T (p.Arg21Cys), has been reported in the cardiac N extension of troponin I by our team,^11^ prompting a series of in vitro and in vivo functional studies of this mutation in recent years.^12–15^

The p.Arg21Cys mutation in *TNNI3* impairs calcium handling and results in an abnormal relaxation of the cardiac sarcomere of mouse models. Functional characterization in vitro and in mouse showed that the cardiac N extension of troponin I serves as a molecular switch. The p.Arg21Cys mutation is located in the RRRSS consensus motif for β_1_-adrenergic-activated PKA (protein kinase A) phosphorylation, and the recombinant cardiac troponin I has decreased phosphorylation by PKA as compared with the wild type.^13^ It also results in increased Ca^2+^ sensitivity of force development during contraction.^13^ The mutation exerts a dominant-negative effect with the mutant cardiac troponin I comprising around 25% of the expression of the protein in knock-in mice,^12^ and it abolishes phosphorylation of 2 adjacent serine residues at positions 23 and 24.^12^ Furthermore, p.Arg21Cys heterozygous mice developed significant degree of hypertrophy, myocyte disarray, and fibrosis.^12^ A more recent molecular phenotyping study of the *TNNI3* p.Arg21Cys knock-in mouse shows that the mutant mice are unable to relax the myofilament through phosphorylation, which results in impaired diastolic function, dysautonomia, and hypertrophy.^15^

Here, we identify the mutation in 5 families from South Lebanon and present phenotypic data on 57 *TNNI3* p.Arg21Cys-related cardiomyopathy patients showing that the *TNNI3* p.Arg21Cys mutation causes a malignant form of HCM characterized by early SCD in most mutation carriers.

## Methods

Methods for this article are detailed in the Data Supplement. The study was approved by the Institutional Review Board at the American University of Beirut and the Partners Human Research Committee, and all subjects signed proper consent and assent forms at recruitment in the study. The authors declare that all supporting data are available within the article.

## Results

### *TNNI3* p.Arg21Cys Is a Common Cause of HCM in Families From South Lebanon

**Figure 1.**
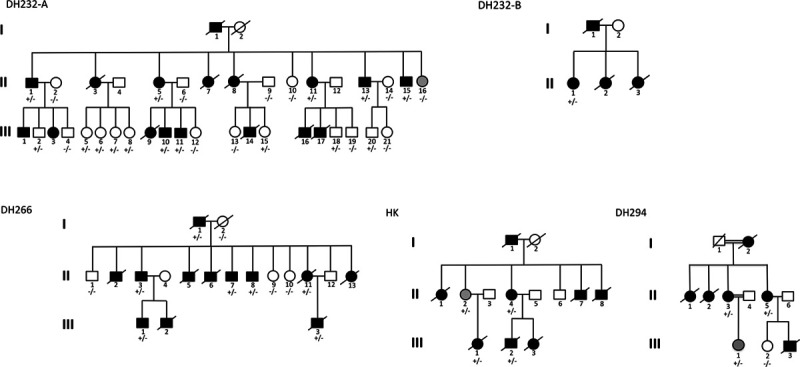
**Pedigrees of families with hypertrophic cardiomyopathy (HCM) due to *TNNI3* p.Arg21Cys.** DH232-A and DH232-B are related; II-1 in DH232-A is first-degree cousin of I-1 in DH232-B. ± denotes the presence of heterozygous *TNNI3* p.Arg21Cys mutation. Circles denote female subjects and boxes, male subjects. Black indicates subjects affected with HCM on echocardiography or subjects who experienced sudden cardiac death, white indicates normal subjects, and gray indicates that the status of the subject is unknown. A slash through the symbol denotes a deceased subject.

We identified a clustering of familial cases of HCM due to *TNNI3* p.Arg21Cys mutation in South Lebanon. Among 29 Lebanese families with HCM, 20 (69%) had at least 1 patient with pediatric (age, <18 years) onset and 7 (24.1%) were from South Lebanon. The *TNNI3* p.Arg21Cys mutation segregated with HCM in 5 families all from South Lebanon and all with at least 1 member with pediatric-onset disease. The logarithm of the odds (LOD) score based on segregation in the 5 families was 4.38 (Figure 1). *TNNI3* p.Arg21Cys explained the phenotype in 17.2% of the total Lebanese cohort and 71.4% of the South Lebanon subset. The likelihood of identifying 5 families in 29 HCM families studied in Lebanon by chance is 5×10^−15^. Only one family (DH294) in this study had 2 consanguineous marriages (Figure 1). Overall, consanguinity was less frequent in this multifamily cohort compared with population estimates in Lebanon.^16^ The mutation was also absent from 2912 sequential HCM patients from a broad referral population who received genetic testing at the Laboratory for Molecular Medicine of Partners Healthcare. In addition, 504 control subjects from Lebanon tested negative for the *TNNI3* p.Arg21Cys mutation (OR, >84; *P*<0.0001), and it was absent from the Genome Aggregation Database, Cambridge, MA (http://gnomad.broadinstitute.org; accessed March 5, 2020), which has 125 748 exomes and 71 702 genomes from unrelated individuals.

Through cascade screening of the 5 families, we identified a total of 57 individuals with *TNNI3* p.Arg21Cys-related cardiomyopathy. Those included 30 confirmed heterozygous carriers of the *TNNI3* p.Arg21Cys mutation and 27 subjects with clinical evidence of HCM—including 22 with SCD in the context of no known medical history—who are implied to have inherited the p.Arg21Cys mutation based on their phenotype and pedigree relationship to a first-degree p.Arg21Cys carrier (Figure 1; Table).

**Table.**
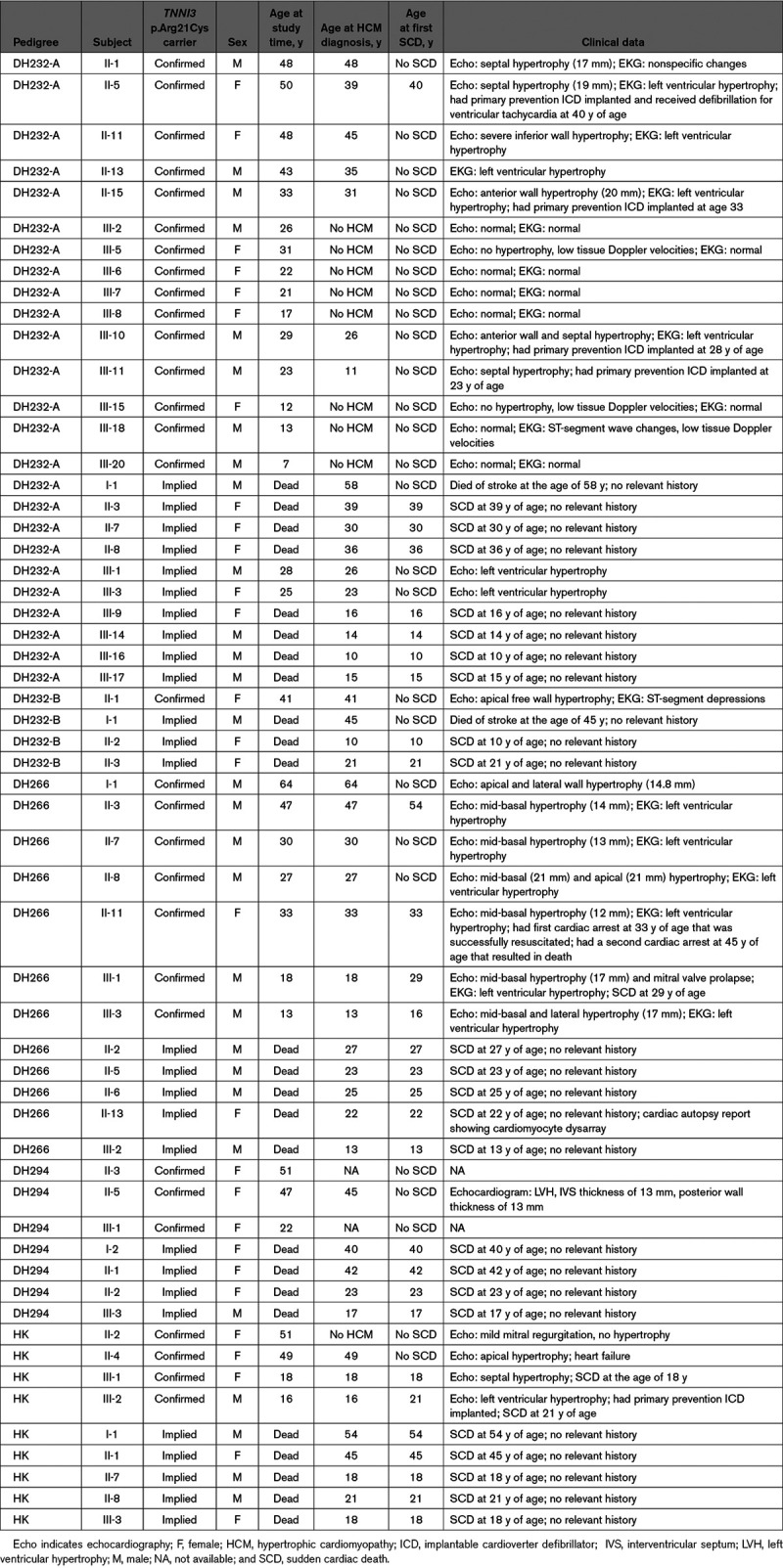
Characteristics of Patients With *TNNI3* p.Arg21Cys-Related Cardiomyopathy

### *TNNI3* p.Arg21Cys-Related Cardiomyopathy Causes Early SCD

**Figure 2.**
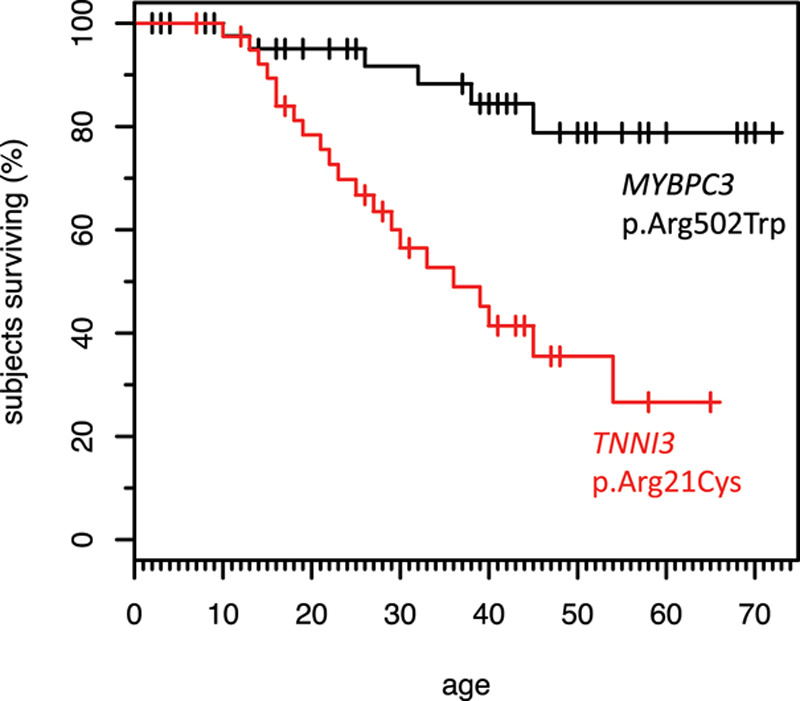
**Survival of subjects with *TNNI3* p.Arg21Cys-related cardiomyopathy compared with carriers of *MYBPC3* p.Arg502Trp.** Age at sudden cardiac death (SCD) for 40 subjects with *TNNI3* p.Arg21Cys-related cardiomyopathy as compared with 47 hypertrophic cardiomyopathy patients with the *MYBPC3* p.Arg502Trp mutation shows the median age of SCD is 22.5 y in the *TNNI* p.Arg21Cys group. At that age, only about 10% of the *MYBC* p.Arg502Trp carriers have SCD.

Patients with *TNNI3* p.Arg21Cys-related cardiomyopathy had a malignant phenotype with frequent SCD at a young age. SCD occurred in 53% (30 of 57) of affected patients at a median age of 22.5 years (interquartile range, 17.2–35.2). SCD was the first presentation of disease in 83.3% (25 of 30) of patients (Table). Survival analysis for 57 *TNNI3* p.Arg21Cys-related cardiomyopathy patients revealed a markedly lower age at the first adverse event as compared with 47 HCM patients with the *MYBPC3* p.Arg502Trp mutation (Figure 2). There were no sex differences in the rates of SCD, 13 of 28 (46.4%) women and 14 of 29 (48.3%) men (*P*=0.89), or the age at SCD in a Cox proportional-hazards model (*P*=0.99).

While most patients had early-onset SCD, 2 patients had stroke (DH232-A I-1 and DH232-B I-1) and 2 other patients (DH232-B II-1 and HK II-4) had the apical variant of HCM. There was no clinical diagnosis of restrictive cardiomyopathy in the cohort.

### SCD in *TNNI3* p.Arg21Cys Carriers Occurs in the Context of Subclinical Disease

Observations on several patients suggested that SCD is occurring despite routine care and disease awareness in the family (Table). One patient (DH266 II-13) had SCD at age 22, 3 months following a normal echocardiogram, and cardiomyocyte disarray was noted on autopsy report. Preclinical disease was also common. Of the 30 carriers with the *TNNI3* p.Arg21Cys mutation, 19 (63.3%) had a clinical diagnosis of HCM based on echocardiography with a median age of 33 years (interquartile range, 22–45), and 9 (30%), with median age 21 years (interquartile range, 13–26), had no evidence of HCM on echocardiography (Table).

**Figure 3.**
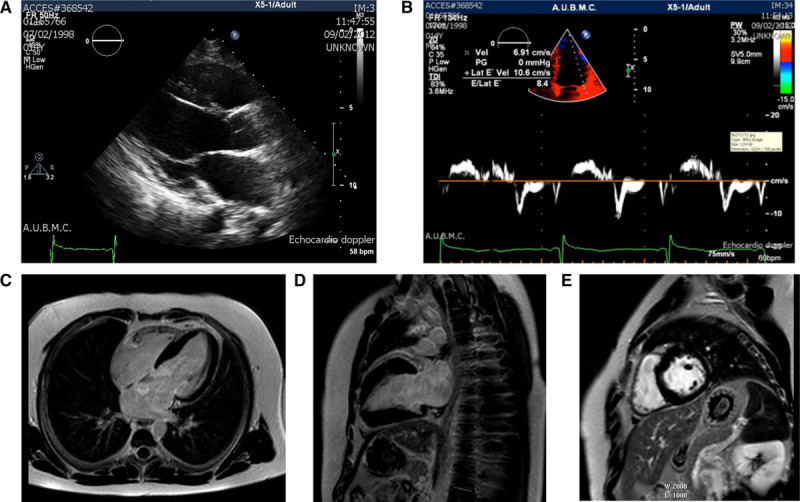
**Advanced cardiac imaging of genotype-positive but phenotype-negative patient.** The 13-y old man (patient III-18 from DH232-A) is asymptomatic and carries the p.Arg21Cys mutation. Cardiac imaging reveals the absence of hypertrophy on echocardiography (**A**); Doppler tissue myocardial velocities at the lateral aspect of the mitral annulus (**B**) show reduced Ea and S velocities, suggesting early systolic and diastolic myocardial dysfunction. Cardiac magnetic resonance images in end diastole (**C–E**) show a relatively asymmetrical wall thickening of the basal and mid-anteroseptal and inferoseptal walls as compared with the lateral wall (10–11 vs 6–7 mm). The wall thickness per se is within normal, and the left ventricular mass total is also normal. There is uniform nulling of the myocardium on delayed imaging post-gadolinium, including the area of maximal thickening. No evidence of scar or fibrosis or infiltrative disease was seen.

**Figure 4.**
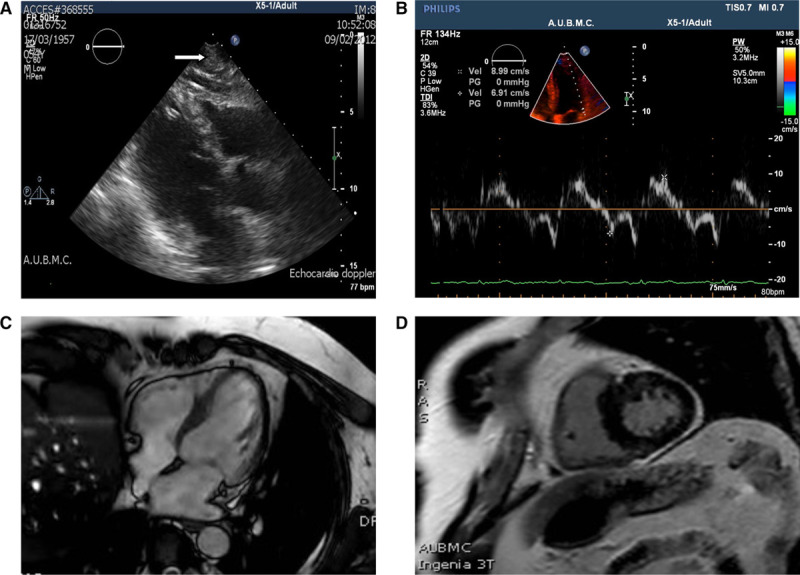
**Advanced cardiac imaging of genotype- and phenotype-positive patient.** The 48-y-old man (patient II-1 from DH232-A) is symptomatic, carries the p.Arg21Cys mutation, and has known left ventricular hypertrophy on echocardiography (arrow; **A**). Doppler tissue myocardial velocities at the lateral aspect of the mitral annulus (**B**) show reduced Ea and S velocities, suggesting early systolic and diastolic myocardial dysfunction. Cardiac magnetic resonance imaging (**C** and **D**) shows septal hypertrophy (13–14 vs 7 mm) at the lateral wall and mild-to-moderate focal enhancement in the mid-anteroseptum consistent with scarring.

To illustrate the importance of identifying subclinical disease in carriers of the *TNNI3* p.Arg21Cys mutation who have no hypertrophy on echocardiogram, we obtained Doppler tissue imaging (DTI) and cardiac magnetic resonance imaging (MRI) on 2 carriers of the mutation, a 13-year-old adolescent (patient III-18 from DH232-A) with no hypertrophy on echocardiogram and, therefore, no clinical diagnosis of HCM before the study, and a 48-year-old man (patient II-1 from DH232-A) with symptomatic HCM (Figures 3 and 4). Following current clinical guidelines without knowledge of the genotype, the adolescent would not have undergone MRI or DTI for screening. His DTI showed reduced Ea and S velocities, suggesting early systolic and diastolic myocardial dysfunction. Cardiac MRI also showed a relatively asymmetrical wall thickening of the basal and mid-anteroseptal and inferoseptal walls as compared with the lateral wall (10–11 versus 6–7 mm), with uniform nulling of the myocardium on delayed imaging post-gadolinium (Figure 3). These findings are similar to the older man with diagnosed left ventricular hypertrophy on echocardiogram who showed also reduced Ea and S velocities on DTI. In addition, the cardiac MRI revealed septal hypertrophy and moderate focal enhancement in the mid-anteroseptum consistent with scarring (Figure 4).

## Discussion

Here, we show that the *TNNI3* p.Arg21Cys mutation has a founder effect in South Lebanon and causes malignant HCM with early SCD even in the absence of hypertrophy. Genetic diagnosis with this mutation may be sufficient for risk stratification for SCD. The phenotype in this multifamily cohort with HCM corroborates the mouse phenotype and the critical role of the N terminus of cardiac troponin to cardiac sarcomere function.

### *TNNI3* p.Arg21Cys Causes Malignant HCM With Early SCD

Carriers of the *TNNI3* p.Arg21Cys mutation were diagnosed with HCM at a younger age than typical HCM patients and had remarkably high rate of SCD, frequently as the first presentation. In current clinical practice, risk stratification for SCD is based on clinical risk factors such as the degree of hypertrophy among others and family history.^6^ With few exceptions, the majority of genotype-phenotype correlations have failed to demonstrate that a gene variant alone could be used to predict risk of sudden death with enough certainty independent of the phenotype.^5,6^ In the specific case of *TNNI3* p.Arg21Cys, our data highlight a malignant phenotype at a young age that justifies risk stratification for SCD based exclusively on genetic information. Mutation carriers may benefit from shared decision-making for implantation of a defibrillator to prevent SCD, even in the absence of any structural abnormality on imaging.

Few observations in our cohort also suggested that SCD could occur in the absence of marked left ventricular hypertrophy on screening echocardiography, which is typically performed in mutation carriers during cascade screening of HCM. Multiple studies have shown that preclinical HCM in mutation carriers could be missed on echocardiogram, while more advanced imaging modalities could detect regional hypertrophy, fibrosis, diastolic dysfunction, or abnormal strain.^17–21^ Advanced imaging modalities such as cardiac MRI could potentially be useful to detect a mild subclinical phenotype in *TNNI* p.Arg21Cys carriers.

### Human HCM Phenotype Corroborates the Critical Role of N Terminus of Cardiac Troponin I

The phenotype of *TNNI3* p.Arg21Cys-related cardiomyopathy in human carriers is consistent with prior studies on *TNNI3* p.Arg21Cys knock-in mouse models. The N terminus of cardiac troponin I consists of a 32-amino-acid extension that is evolutionarily conserved and unique to cardiac troponin I (missing from skeletal isoforms).^14^ Functionally, this region has been associated directly to the binding of PKA to a consensus RRRSS sequence upon β-adrenergic stimulation of the heart mainly through the phosphorylation of 2 serine residues at positions 23 and 24 of cardiac troponin I.^14^ In the knock-in mouse with the p.Arg21Cys mutation, the PKA-mediated phosphorylation of those residues secondary to β-adrenergic stimulation is abolished.^12^ As a result, there is impaired lusitropy or relaxation of the cardiac myofilament.^15^ This impaired myofilament relaxation kinetics predisposes the heart to abnormal diastolic dysfunction especially during periods of β-adrenergic stimulation such as strenuous physical activity, potentially precipitating arrhythmias.^15^ In the families we describe, several young carriers of *TNNI3* p.Arg21Cys presented with story of SCD during bouts of physical activity, consistent with this physiological mechanism described in the mouse model. While current medical treatments in HCM are exclusively geared at improving symptoms in obstructive disease, pilot studies have shown a potential role for calcium channel inhibition in improving early left ventricular modeling in preclinical disease, but this area requires further research.^22^

### Limitations

This study has several limitations. First, we only performed targeted testing for subclinical phenotype on few participants while the remainder of the clinical data were obtained as part of routine medical care. Second, because participants were recruited based on cascade screening, there was a heterogeneity in the way clinical care was delivered by multiple providers, and we are unable to consistently evaluate guideline-based clinical risk stratification for SCD in all participants. Third, a large portion of the study participants experienced SCD before genotyping or even seeking medical care, making it impossible to definitely exclude other causes of death, although this is unlikely. Fourth, while we investigated the occurrence of SCD in carriers, we did not systematically evaluate the impact of the mutation on other common sequelae in HCM such as heart failure, stroke, and atrial fibrillation. Fifth, we observe significant variability in incidence of SCD even among members of the same family, which raises the possibility of monogenic, polygenic, or nongenetic modifiers of the phenotype.

### Conclusions

*TNNI3* p.Arg21Cys-related cardiomyopathy has a founder effect in South Lebanon and causes a malignant phenotype characterized by early-onset SCD, sometimes in the absence of significant hypertrophy. Phenotypic findings in patients with the mutation corroborate the important molecular role of the N terminus of cardiac troponin I in calcium handling and diastolic relaxation of the cardiac sarcomere. Genetically informed risk stratification and management for prevention of SCD is challenging due to incomplete penetrance and variable expressivity, but this rare mutation is one example where this might be possible.

## Acknowledgments

We would like to thank the patients and families for their participation in the study.

## Sources of Funding

This study was funded by the Dubai Harvard Foundation for Medical Research (Drs Fahed, Nemer, Bitar, J.G. Seidman, and C.E. Seidman), the National Institutes of Health (Drs Fahed, J.G. Seidman, and C.E. Seidman), the Howard Hughes Medical Institute (C.E. Seidman), and the Wellcome Trust (107469/Z/15/Z; Dr Ware).

## Disclosures

Dr Fahed is a consultant and owns shares in Goodpath, which was not involved in the study. Dr Ware reports receiving grants and personal fees from MyoKardia outside the submitted work. Drs J.G. Seidman and C.E. Seidman are founders and own shares in Myokardia—a company that is developing therapeutics that target the sarcomere, which was not involved in this study. The other authors report no conflicts.

## Supplementary Material



## References

[1] Seidman CE Seidman JG Identifying sarcomere gene mutations in hypertrophic cardiomyopathy: a personal history. Circ Res. 2011;108:743–750. doi: 10.1161/CIRCRESAHA.110.223834 21415408 10.1161/CIRCRESAHA.110.223834 PMC3072749

[2] Ho CY Day SM Ashley EA Michels M Pereira AC Jacoby D Cirino AL Fox JC Lakdawala NK Ware JS Genotype and lifetime burden of disease in hypertrophic cardiomyopathy: insights from the Sarcomeric Human Cardiomyopathy Registry (SHaRe). Circulation. 2018;138:1387–1398. doi: 10.1161/CIRCULATIONAHA.117.033200 30297972 10.1161/CIRCULATIONAHA.117.033200 PMC6170149

[3] Weissler-Snir A Allan K Cunningham K Connelly KA Lee DS Spears DA Rakowski H Dorian P Hypertrophic cardiomyopathy-related sudden cardiac death in young people in Ontario. Circulation. 2019;140:1706–1716. doi: 10.1161/CIRCULATIONAHA.119.040271 31630535 10.1161/CIRCULATIONAHA.119.040271

[4] Jayaraman R Reinier K Nair S Aro AL Uy-Evanado A Rusinaru C Stecker EC Gunson K Jui J Chugh SS Risk factors of sudden cardiac death in the young: multiple-year community-wide assessment. Circulation. 2018;137:1561–1570. doi: 10.1161/CIRCULATIONAHA.117.031262 29269388 10.1161/CIRCULATIONAHA.117.031262 PMC5918307

[5] Tower-Rader A Desai MY Phenotype-genotype correlation in hypertrophic cardiomyopathy: less signal, more noise? Circ Cardiovasc Imaging. 2017;10:e006066 28193615 10.1161/CIRCIMAGING.117.006066

[6] Mogensen J Murphy RT Kubo T Bahl A Moon JC Klausen IC Elliott PM McKenna WJ Frequency and clinical expression of cardiac troponin I mutations in 748 consecutive families with hypertrophic cardiomyopathy. J Am Coll Cardiol. 2004;44:2315–2325. doi: 10.1016/j.jacc.2004.05.088 15607392 10.1016/j.jacc.2004.05.088

[7] Maron MS Rowin EJ Wessler BS Mooney PJ Fatima A Patel P Koethe BC Romashko M Link MS Maron BJ Enhanced American College of Cardiology/American Heart Association strategy for prevention of sudden cardiac death in high-risk patients with hypertrophic cardiomyopathy. JAMA Cardiol. 2019;4:644–657. doi: 10.1001/jamacardio.2019.1391 31116360 10.1001/jamacardio.2019.1391 PMC6537832

[8] Gersh BJ Maron BJ Bonow RO Dearani JA Fifer MA Link MS Naidu SS Nishimura RA Ommen SR Rakowski H; American College of Cardiology Foundation/American Heart Association Task Force on Practice Guidelines; American Association for Thoracic Surgery; American Society of Echocardiography; American Society of Nuclear Cardiology; Heart Failure Society of America; Heart Rhythm Society; Society for Cardiovascular Angiography and Interventions; Society of Thoracic Surgeons. 2011 ACCF/AHA guideline for the diagnosis and treatment of hypertrophic cardiomyopathy: executive summary: a report of the American College of Cardiology Foundation/American Heart Association Task Force on practice guidelines. Circulation. 2011;124:2761–2796. doi: 10.1161/CIR.0b013e318223e230 22068435 10.1161/CIR.0b013e318223e230

[9] Murphy RT Mogensen J Shaw A Kubo T Hughes S McKenna WJ Novel mutation in cardiac troponin I in recessive idiopathic dilated cardiomyopathy. Lancet. 2004;363:371–372. doi: 10.1016/S0140-6736(04)15468-8 15070570 10.1016/S0140-6736(04)15468-8

[10] Mogensen J Kubo T Duque M Uribe W Shaw A Murphy R Gimeno JR Elliott P McKenna WJ Idiopathic restrictive cardiomyopathy is part of the clinical expression of cardiac troponin I mutations. J Clin Invest. 2003;111:209–216. doi: 10.1172/JCI16336 12531876 10.1172/JCI16336 PMC151864

[11] Arad M Penas-Lado M Monserrat L Maron BJ Sherrid M Ho CY Barr S Karim A Olson TM Kamisago M Gene mutations in apical hypertrophic cardiomyopathy. Circulation. 2005;112:2805–2811. doi: 10.1161/CIRCULATIONAHA.105.547448 16267253 10.1161/CIRCULATIONAHA.105.547448

[12] Wang Y Pinto JR Solis RS Dweck D Liang J Diaz-Perez Z Ge Y Walker JW Potter JD Generation and functional characterization of knock-in mice harboring the cardiac troponin I-R21C mutation associated with hypertrophic cardiomyopathy. J Biol Chem. 2012;287:2156–2167. doi: 10.1074/jbc.M111.294306 22086914 10.1074/jbc.M111.294306 PMC3265894

[13] Gomes AV Harada K Potter JD A mutation in the N-terminus of troponin I that is associated with hypertrophic cardiomyopathy affects the Ca(2+)-sensitivity, phosphorylation kinetics and proteolytic susceptibility of troponin. J Mol Cell Cardiol. 2005;39:754–765. doi: 10.1016/j.yjmcc.2005.05.013 16005017 10.1016/j.yjmcc.2005.05.013

[14] Howarth JW Meller J Solaro RJ Trewhella J Rosevear PR Phosphorylation-dependent conformational transition of the cardiac specific N-extension of troponin I in cardiac troponin. J Mol Biol. 2007;373:706–722. doi: 10.1016/j.jmb.2007.08.035 17854829 10.1016/j.jmb.2007.08.035

[15] Dweck D Sanchez-Gonzalez MA Chang AN Dulce RA Badger CD Koutnik AP Ruiz EL Griffin B Liang J Kabbaj M Long term ablation of protein kinase A (PKA)-mediated cardiac troponin I phosphorylation leads to excitation-contraction uncoupling and diastolic dysfunction in a knock-in mouse model of hypertrophic cardiomyopathy. J Biol Chem. 2014;289:23097–23111. doi: 10.1074/jbc.M114.561472 24973218 10.1074/jbc.M114.561472 PMC4132808

[16] Barbour B Salameh P Consanguinity in Lebanon: prevalence, distribution and determinants. J Biosoc Sci. 2009;41:505–517. doi: 10.1017/S0021932009003290 19175949 10.1017/S0021932009003290

[17] Valente AM Lakdawala NK Powell AJ Evans SP Cirino AL Orav EJ MacRae CA Colan SD Ho CY Comparison of echocardiographic and cardiac magnetic resonance imaging in hypertrophic cardiomyopathy sarcomere mutation carriers without left ventricular hypertrophy. Circ Cardiovasc Genet. 2013;6:230–237. doi: 10.1161/CIRCGENETICS.113.000037 23690394 10.1161/CIRCGENETICS.113.000037 PMC3974911

[18] Hiremath P Lawler PR Ho JE Correia AW Abbasi SA Kwong RY Jerosch-Herold M Ho CY Cheng S Ultrasonic assessment of myocardial microstructure in hypertrophic cardiomyopathy sarcomere mutation carriers with and without left ventricular hypertrophy. Circ Heart Fail. 2016;9:e003026 27623770 10.1161/CIRCHEARTFAILURE.116.003026 PMC5024718

[19] Vigneault DM Yang E Jensen PJ Tee MW Farhad H Chu L Noble JA Day SM Colan SD Russell MW Left ventricular strain is abnormal in preclinical and overt hypertrophic cardiomyopathy: cardiac MR feature tracking. Radiology. 2019;290:640–648. doi: 10.1148/radiol.2018180339 30561279 10.1148/radiol.2018180339 PMC6394738

[20] Maron MS Maron BJ Harrigan C Buros J Gibson CM Olivotto I Biller L Lesser JR Udelson JE Manning WJ Hypertrophic cardiomyopathy phenotype revisited after 50 years with cardiovascular magnetic resonance. J Am Coll Cardiol. 2009;54:220–228. doi: 10.1016/j.jacc.2009.05.006 19589434 10.1016/j.jacc.2009.05.006

[21] Ho CY López B Coelho-Filho OR Lakdawala NK Cirino AL Jarolim P Kwong R González A Colan SD Seidman JG Myocardial fibrosis as an early manifestation of hypertrophic cardiomyopathy. N Engl J Med. 2010;363:552–563. doi: 10.1056/NEJMoa1002659 20818890 10.1056/NEJMoa1002659 PMC3049917

[22] Ho CY Lakdawala NK Cirino AL Lipshultz SE Sparks E Abbasi SA Kwong RY Antman EM Semsarian C González A Diltiazem treatment for pre-clinical hypertrophic cardiomyopathy sarcomere mutation carriers: a pilot randomized trial to modify disease expression. JACC Heart Fail. 2015;3:180–188. doi: 10.1016/j.jchf.2014.08.003 25543971 10.1016/j.jchf.2014.08.003 PMC4323670

