# Supplementary material for: Founder Mutation in N Terminus of Cardiac Troponin I Causes Malignant Hypertrophic Cardiomyopathy
Source: Circ Genom Precis Med. 2020 Sep 4;13(5):444–52. doi: 10.1161/CIRCGEN.120.002991 (PMC7676616; doi:10.1161/CIRCGEN.120.002991)
Supplement: Supplementary file 1 [file hcg-13-444-s001.pdf]

Supplemental Material

Methods

Subject recruitment and ascertainment of the phenotype

We recruited twenty-nine consecutive families with HCM from patients presenting to cardiology clinics at the American University of Beirut Medical Center in Lebanon. All index patients underwent routine clinical evaluation, review of medical records, electrocardiogram, and echocardiogram similar to previous familial studies.[1-3] Following recruitment of the index patient in a clinic setting, we performed cascade screening to recruit family members through community visits. We interviewed family members to obtain pedigree data including consanguinity, history of sudden cardiac death events, reviewed existing medical records, and in selected cases invited individuals for additional clinical evaluation similar to the index patient. We performed echocardiographic doppler tissue imaging and cardiac magnetic resonance imaging on selected patients. All subjects provided a sample of peripheral blood for DNA extraction.

We also recruited 504 Lebanese control subjects without any history of cardiac disease, including 117 young healthy controls and 387 unrelated parents of patients with congenital heart disease.

Genetic testing and analysis

We extracted genomic DNA for all patients, family members and normal controls from peripheral blood using a commercial kit from QIAGEN. Initially we performed targeted sequencing for a custom sub-genome containing the exons of 68 candidate genes implicated in

heart muscle disease, including six definite HCM genes (*MYH7*, *MYBPC3*, *TNNI3*, *ACTC1*, *TNNT2*, and *TPM1*) and ten posited HCM genes (*ACTN2*, *ANKRD1*, *CSRP3*, *LDB3*, *MYH6*, *PLN*, *TCAP*, *TNNC1*, *TTN*, and *VCL*) on three subjects from family DH232-A (II-5, III-11, and II-15) (Figure 1). Briefly, we constructed DNA libraries constructed from genomic DNA, and in-solution capture was carried using the SureSelect Target Enrichment system from Agilent and following the manufacturer's protocol (Agilent SureSelect Target Enrichment; Agilent, Santa Rosa, CA). An Illumina HiSeq 2000 platform was used for paired-end sequencing of the captured libraries. Next-generation sequence data was analyzed as described previously.[4, 5]

After confirming the *TNNI3* p.Arg21Cys mutation in the DH232-A family, we performed Sanger sequencing to identify the mutation in the remaining families. We designed primers (forward primer, 5' GTA CTC TGC CCC CAG GAA G 3'; reverse primer, 5' AGA GGG TGC GGT ACG GTA AG 3') to amplify a 184bp DNA fragment harboring the p.Arg21Cys mutation. PCR-amplified fragments were purified and sequenced on an ABI PRISM BigDye terminator v3.1 ready reaction cycle sequencing kit (catalog # 4337454, Applied Biosystems, Waltham, MA). To screen for the p.Arg21Cys mutation in our familial cohort, we performed Sanger sequencing on at least one HCM patient from the remaining 28 families. This identified the presence of *TNNI3* p.Arg21Cys in at least one HCM patient in another 4 families (DH232B, DH266, DH294, and HK). We then sequenced all members of the five families with available DNA (Figure 1). To confirm segregation of *TNNI3* p.Arg21Cys with HCM in each of the five families, we calculated logarithm of odds (LOD) scores using Fastlink, assuming autosomal dominant inheritance, penetrance of 90%, disease gene frequency of 0.001 and theta of zero.

In order to ensure the variant is not seen in unaffected individuals from south Lebanon, we performed genotyping of 504 control subjects using a MassArray system (Sequenom

iPLEXassay, San Diego, USA). In brief, genomic DNA was amplified using a locus-specific PCR and detection primer, and then the PCR products were used for locus-specific single-base extension reaction, after which the alleles were discriminated by mass spectrometry. In addition to the 504 controls subjects, all family members carrying the p.Arg21Cys mutation as determined by sequencing were confirmed in the MassArray genotyping experiment as positive controls.

Sudden cardiac death

We defined the outcome of sudden cardiac death as one of the following: sudden unexplained death occurring during physical exertion in an otherwise healthy person, sustained ventricular tachycardia or ventricular fibrillation confirmed on electrocardiogram or on a recording of an implantable defibrillator device, or autopsy report confirming HCM as the cause of death. The age at sudden cardiac death was modeled using Kaplan-Meier survival analysis with censorship at age of occurrence of SCD data on all 57 subjects with *TNNI3* p.Arg21Cys-related cardiomyopathy. Comparison of survival data was made on SCD events, defined similarly, in 47 HCM patients with confirmed p.Arg502Trp *MYBPC3* mutation available through our lab. Statistical analyses were performed with the use of R software, version 3.4 (R Project for Statistical Computing).

References:

1. Niimura H, Bachinski LL, Sangwatanaroj S, Watkins H, Chudley AE, McKenna W, Kristinsson A, Roberts R, Sole M, Maron BJ, et al. Mutations in the gene for cardiac myosin-binding protein C and late-onset familial hypertrophic cardiomyopathy. *NEJM*. 1998;338:1248-57.

2. Niimura H, Patton KK, McKenna WJ, Soults J, Maron BJ, Seidman JG, Seidman CE. Sarcomere protein gene mutations in hypertrophic cardiomyopathy of the elderly. *Circulation*. 2002;105:446-51.

3. Maron BJ, Niimura H, Casey SA, Soper MK, Wright GB, Seidman JG, Seidman CE. Development of left ventricular hypertrophy in adults in hypertrophic cardiomyopathy caused by cardiac myosin-binding protein C gene mutations. *JACC*. 2001;38:315-21.

4. Abou Hassan OK, Fahed AC, Batrawi M, Arabi M, Refaat MM, DePalma SR, Seidman JG, Seidman CE, Bitar FF, Nemer GM. NKX2-5 mutations in an inbred consanguineous population: genetic and phenotypic diversity. *Sci Rep.* 2015;5:8848.

5. Herman DS, Lam L, Taylor MR, Wang L, Teekakirikul P, Christodoulou D, Conner L, DePalma SR, McDonough B, Sparks E, et al. Truncations of titin causing dilated cardiomyopathy. *NEJM*. 2012;366:619-28.
