# Supplementary material for: Founder Mutation in N Terminus of Cardiac Troponin I Causes Malignant Hypertrophic Cardiomyopathy
Source: Circ Genom Precis Med. 2020 Sep 4;13(5):444–52. doi: 10.1161/CIRCGEN.120.002991 (PMC7676616; doi:10.1161/CIRCGEN.120.002991)
Supplement: Supplementary file 2 [file hcg-13-444-s002.pdf]

Founder Mutation in N-terminus of Cardiac Troponin I Causes Malignant Hypertrophic Cardiomyopathy

Running title: *Fahed & Nemer et al.; Malignant HCM due to TNNI3 p.Arg21Cys*

Akl C. Fahed, MD, MPH[1,2]*; Georges Nemer, PhD[3,4]*; Fadi F. Bitar, MD[3,5]; Samir Arnaout, MD[6]; Antoine B. Abchee, MD[6]; Manal Batrawi, MS[3]; Athar Khalil, PhD[3]; Oussama Abou-Hassan, MD[3,6]; Steven R. DePalma, PhD[7]; Barbara McDonough, RN[7]; Mariam T. Arabi, MD[5]; James S. Ware, PhD, MRCP[8,9]; Jonathan G. Seidman, PhD[7]; Christine E. Seidman, MD[7,10]

[1]Center of Genomic Medicine & Division of Cardiology, Dept of Medicine, Massachusetts General Hospital, [7]Dept of Genetics, Harvard Medical School, Boston; [2]Cardiovascular Disease Initiative, Broad Inst of MIT & Harvard, Cambridge, MA; [3]Dept of Biochemistry & Molecular Genetics, American Univ of Beirut, Beirut, Lebanon; [4]College of Health & Life Sciences, Hamad Bin Khalifa Univ Education City, Doha, Qatar; [5]Dept of Pediatrics, [6]Cardiology Division, American Univ of Beirut Medical Center, Beirut, Lebanon; [8]National Heart & Lung Institute, Imperial College London, Royal Brompton Hospital; [9]Medical Research College (MRC) London Inst of Medical Sciences, London, UK; [10]Division of Cardiology & Howard Hughes Medical Inst, Brigham and Women's Hospital, Boston, MA
*contributed equally

Circulation: Genomic and Precision Medicine

Correspondence:

Jonathan G. Seidman, MD
Dept of Genetics, NRB Room 256
Harvard Medical School
77 Avenue Louis Pasteur
Boston, MA 02115
Tel: 617-432-7871
Email: seidman@genetics.med.harvard.edu

Christine E Seidman, MD
Dept of Genetics, NRB 257
Harvard Medical School
77 Avenue Louis Pasteur
Boston, MA 02115
Tel: 617-432-7871
Email: cseidman@genetics.med.harvard.edu

Journal Subject Terms: Genetics; Heart Failure; Cardiomyopathy

Abstract

Background - Cardiac troponin I (*TNNI3*) gene mutations account for 3% of hypertrophic cardiomyopathy and carriers have a heterogeneous phenotype, with increased risk of sudden cardiac death. Only one mutation (p.Arg21Cys) has been reported in the N-terminus of the protein. In model organisms, it impairs protein kinase A phosphorylation, increases calcium sensitivity, and causes diastolic dysfunction. The phenotype of this unique mutation in hypertrophic cardiomyopathy patients remains unknown.

Methods - We sequenced 29 families with hypertrophic cardiomyopathy enriched for pediatric-onset disease and identified 5 families with the *TNNI3* p.Arg21Cys mutation. Using cascade screening, we studied the clinical phenotype of 57 individuals from the 5 families with *TNNI3* p.Arg21Cys-related cardiomyopathy. We performed survival analysis investigating the age at first sudden cardiac death in carriers of the mutation.

Results - All five families with *TNNI3* p.Arg21Cys were from south Lebanon. *TNNI3* p.Arg21Cys-related cardiomyopathy manifested a malignant phenotype – sudden cardiac death occurred in 30 (53%) of 57 affected individuals at median age of 22.5 years. In select carriers without left ventricular hypertrophy on echocardiogram, sudden cardiac death occurred, myocyte disarray was found on autopsy heart, and tissue doppler and cardiac magnetic resonance imaging identified subclinical disease features such as diastolic dysfunction and late-gadolinium enhancement.

Conclusions - The *TNNI3* p.Arg21Cys mutation has a founder effect in south Lebanon and causes malignant hypertrophic cardiomyopathy with early sudden cardiac death even in the absence of hypertrophy. Genetic diagnosis with this mutation may be sufficient for risk stratification for sudden cardiac death.

American Heart Association.
Circulation: Genomic and Precision Medicine

Key words: hypertrophic cardiomyopathy; sudden cardiac death; cardiac troponin I; founder mutation

Nonstandard Abbreviations and Acronyms

TNNI3	Cardiac troponin I gene
SCD	Sudden cardiac death
MRI	Magnetic resonance imaging
HCM	Hypertrophic cardiomyopathy

Introduction

Hypertrophic cardiomyopathy (HCM) is a disease of cardiac muscle caused by sarcomere gene mutations and is associated with increased risk of sudden cardiac death.[1, 2] Among patients 10-45 years of age, sudden cardiac death occurs at an annual incidence of less than 1 in a 1000, with the majority occurring in previously undiagnosed individuals.[3] Out of all sudden cardiac death cases in people aged 5-34 years, 14% are due to hypertrophic cardiomyopathy.[4] The weak genotype-phenotype correlations and wide phenotypic variability of the disease within and between families limit the ability of using genetics to predict who will experience sudden cardiac death.[5,6] For example, while one HCM patient could have unexplained asymmetric left ventricular hypertrophy at a young age and subsequently experience sudden cardiac death, many others have subclinical disease and remain undiagnosed unless detected by genetic screening, often performed after a family member is diagnosed.

Circulation: Genomic and Precision Medicine

An understanding of the phenotype driven by a specific gene mutation with known molecular mechanism could provide an opportunity for more personalized treatment of HCM. If a molecular genotype predicts substantial risk of sudden cardiac death (SCD), then carriers can place an implantable cardioverter defibrillator – a very effective strategy for prevention of sudden cardiac death in HCM patients.[7] Currently, implanting a defibrillator is based on clinical criteria such as presence of ventricular arrhythmias, syncope or prior cardiac arrest, family history of a close relative with SCD, and massive myocardial thickness.[8] With few exceptions,

using genetic mutations to inform risk stratification for sudden cardiac death in HCM patients is limited for three reasons. First, most mutations are private precluding the availability of large cohorts with a single mutation. Second, mutations in the same gene can have differential effects on the protein structure and subsequently the phenotype. Third, clinical phenotypes such as cardiac hypertrophy are the end result of different molecular pathways.

Mutations in the gene encoding the cardiac troponin I (*TNNI3)* account to around 3% of HCM and also have a heterogeneous phenotype.[6] More than 55 mutations in *TNNI3* have been reported to cause cardiomyopathy, mostly hypertrophic, but also a minority that can cause a dilated or restrictive cardiomyopathy.[9,10] Troponin is a protein complex made of troponin I, troponin C, and troponin T and is located within the thin filament of the sarcomere where it is responsible for binding calcium and switching contraction. Upon calcium binding, troponin undergoes a series of conformational changes allowing the release of troponin I inhibition from actin and resulting in actin-myosin binding and force generation. The cardiac troponin I is different from skeletal troponin I in that it has an additional 32 amino-acid sequence on its N-terminus domain. Only one HCM causing mutation, NM_000363.5:c.61C>T (p.Arg21Cys), has been reported in the cardiac N-extension of troponin I by our team,[11] prompting a series of *in-vitro* and *in-vivo* functional studies of this mutation in recent years.[12-15]

American Heart Association.
Circulation: Genomic and Precision Medicine

The p.Arg21Cys mutation in *TNNI3* impairs calcium handling and results in an abnormal relaxation of the cardiac sarcomere of mouse models. Functional characterization *in-vitro* as well as in mouse showed that the cardiac N-extension of troponin I serves as a molecular switch. The p.Arg21Cys mutation is located in the "RRRSS" consensus motif for β_1–adrenergic-activated protein kinase A (PKA) phosphorylation, and the recombinant cardiac troponin I has decreased phosphorylation by PKA as compared to the wild-type.[13] It also results in increased Ca^{2+}-

sensitivity of force development during contraction.[13] The mutation exerts a dominant-negative effect with the mutant cardiac troponin I comprising around 25% of the expression of the protein in knock-in mice,[12] and it abolishes phosphorylation of two adjacent serine residues at positions 23 and 24.[12] Furthermore, p.Arg21Cys heterozygous mice developed significant degree of hypertrophy, myocyte disarray, and fibrosis.[12] A more recent molecular phenotyping study of the *TNNI3* p.Arg21Cys knock-in mouse shows that the mutant mice are unable to relax the myofilament through phosphorylation, which results in impaired diastolic function, dysautonomia, and hypertrophy.[15]

Here, we identify the mutation in five families from south Lebanon and present phenotypic data on 57 *TNNI3* p.Arg21Cys-related cardiomyopathy patients showing that the *TNNI3* p.Arg21Cys mutation causes a malignant form of HCM characterized by early sudden cardiac death in most mutation carriers.

Circulation: Genomic and Precision Medicine

Methods

Methods for this paper are detailed in the online supplemental material. The study was approved by the Institutional Review Board at the American University of Beirut and the Partners Human Research Committee, and all subjects signed proper consent and assent forms at recruitment in the study. The authors declare that all supporting data are available within the article.

Results

***TNNI3* p.Arg21Cys is a common cause of HCM in families from south Lebanon**

We identified a clustering of familial cases of HCM due to *TNNI3* p.Arg21Cys mutation in south Lebanon. Among 29 Lebanese families with HCM, 20 (69%) had at least one patient with

pediatric (age <18 years) onset, and 7 (24.1%) were from south Lebanon. The *TNNI3* p.Arg21Cys mutation segregated with HCM in 5 families all from south Lebanon and all with at least one member with pediatric onset disease. The LOD score based on segregation in the 5 families was 4.38. (Figure 1). *TNNI3* p.Arg21Cys explained the phenotype in 17.2% of the total Lebanese cohort and 71.4% of the south Lebanon subset. The likelihood of identifying 5 families in 29 HCM families studied in Lebanon by chance is 5e-15. Only one family (DH-294) in this study had two consanguineous marriages (Figure 1). Overall, consanguinity was less frequent in this multifamily cohort compared to population estimates in Lebanon.[16] The mutation was also absent from 2,912 sequential HCM patients from a broad referral population who received genetic testing at the Laboratory for Molecular Medicine of Partners Healthcare. In addition, 504 control subjects from Lebanon tested negative for the *TNNI3* p.Arg21Cys mutation (OR>84, p-value<0.0001), and it was absent from the Genome Aggregation Database (gnomAD), Cambridge, MA (URL: http://gnomad.broadinstitute.org; accessed March 5, 2020), which has 125,748 exomes and 71,702 genomes from unrelated individuals.

American Heart Association.
Circulation: Genomic and Precision Medicine

Through cascade screening of the 5 families, we identified a total of 57 individuals with *TNNI3* p.Arg21Cys-related cardiomyopathy. Those included 30 confirmed heterozygous carriers of the *TNNI3* p.Arg21Cys mutation and 27 subjects with clinical evidence of HCM – including 22 with sudden cardiac death in the context of no known medical history – who are implied to have inherited the p.Arg21Cys mutation based on their phenotype and pedigree relationship to a first-degree p.Arg21Cys carrier (Figure 1, Table 1).

TNNI3 p.Arg21Cys-related cardiomyopathy causes early sudden cardiac death

Patients with *TNNI3* p.Arg21Cys-related cardiomyopathy had a malignant phenotype with frequent sudden cardiac death at a young age. Sudden cardiac death occurred in 53% (30/57) of

affected patients at median age of 22.5 years [IQR 17.2-35.2]. SCD was the first presentation of disease in 83.3% (25/30) of patients (Table 1). Survival analysis for 57 *TNNI3* p.Arg21Cys-related cardiomyopathy patients revealed a markedly lower age at first adverse event as compared to 47 HCM patients with the *MYBPC3* p.Arg502Trp mutation (Figure 2). There were no sex differences in the rates of sudden cardiac death – 13/28 (46.4%) of females and 14/29 (48.3%) of males (p=0.89) – or the age at sudden cardiac death in a cox proportional-hazards model (p=0.99).

While most patients had early onset SCD, two patients had stroke (DH232-A I-1 and DH232-B I-1) and two other patients (DH232B II-1 and HK II-4) had the apical variant of HCM. There was no clinical diagnosis of restrictive cardiomyopathy (RCM) in the cohort.

American Heart Association.

Sudden cardiac death in *TNNI3* p.Arg21Cys carriers occurs in the context of subclinical disease

Circulation: Genomic and Precision Medicine

Observations on several patients suggested that sudden cardiac death is occurring despite routine care and disease awareness in the family (Table 1). One patient (DH266 II-13) had SCD at age 22, three months following a normal echocardiogram, and cardiomyocyte disarray was noted on autopsy report. Pre-clinical disease was also common. Out of the 30 carriers with the *TNNI3* p.Arg21Cys mutation, 19 (63.3%) had a clinical diagnosis of HCM based on echocardiography with a median age of 33 years (IQR 22-45], and 9 (30%), with median age 21 years [IQR 13-26], had no evidence of HCM on echocardiography (Table 1).

To illustrate the importance of identifying subclinical disease in carriers of the *TNNI3* p.Arg21Cys mutation who have no hypertrophy on echocardiogram, we obtained doppler tissue imaging and cardiac MRI on two carriers of the mutation, a 13-year old adolescent (patient III-18 from DH232-A) with no hypertrophy on echocardiogram and therefore no clinical diagnosis of

HCM prior to the study, and a 48-year old man (patient II-1 from DH232-A) with symptomatic HCM (Figures 3 and 4). Following current clinical guidelines without knowledge of the genotype, the adolescent would not have undergone MRI or tissue doppler imaging for screening. His doppler tissue imaging showed reduced Ea and S velocities, suggesting early systolic and diastolic myocardial dysfunction. Cardiac MRI also showed a relatively asymmetrical wall thickening of the basal and mid antero-septal and infero-septal walls as compared to the lateral wall (10-11 mm vs 6-7 mm), with uniform nulling of the myocardium on delayed imaging post Gadolinium (Figure 3). These findings are similar to the older man with diagnosed left ventricular hypertrophy on echocardiogram who showed also reduced Ea and S velocities on DTI. In addition, the cardiac MRI revealed septal hypertrophy and moderate focal enhancement in the mid antero-septum consistent with scarring (Figure 4).

American Heart Association.
Circulation: Genomic and Precision Medicine

Discussion

Here, we show that the *TNNI3* p.Arg21Cys mutation has a founder effect in south Lebanon and causes malignant hypertrophic cardiomyopathy with early sudden cardiac death even in the absence of hypertrophy. Genetic diagnosis with this mutation may be sufficient for risk stratification for sudden cardiac death. The phenotype in this multi-family cohort with HCM corroborates the mouse phenotype and the critical role of the N-terminus of cardiac troponin to cardiac sarcomere function.

***TNNI3* p.Arg21Cys causes malignant HCM with early sudden cardiac death**

Carriers of the *TNNI3* p.Arg21Cys mutation were diagnosed with HCM at a younger age than typical HCM patients and had remarkably high rate of sudden cardiac death, frequently as the first presentation. In current clinical practice, risk stratification for sudden cardiac death is based

on clinical risk factors such as the degree of hypertrophy among others, and family history.[6] With few exceptions, the majority of genotype-phenotype correlations have failed to demonstrate that a gene variant alone could be used to predict risk of sudden death with enough certainty independent of the phenotype.[5, 6] In the specific case of *TNNI3* p.Arg21Cys, our data highlight a malignant phenotype at a young age that justifies risk stratification for sudden cardiac death based exclusively on genetic information. Mutation carriers may benefit from shared decision making for implantation of a defibrillator to prevent sudden cardiac death, even in the absence of any structural abnormality on imaging.

Few observations in our cohort also suggested that sudden cardiac death could occur in the absence of marked left ventricular hypertrophy on screening echocardiography, which is typically performed in mutation carriers during cascade screening of HCM. Multiple studies have shown that pre-clinical HCM in mutation carriers could be missed on echocardiogram, while more advanced imaging modalities could detect regional hypertrophy, fibrosis, diastolic dysfunction, or abnormal strain.[17-21] Advanced imaging modalities such as cardiac MRI could potentially be useful to detect a mild subclinical phenotype in *TNNI* p.Arg21Cys carriers.

American Heart Association.
Circulation: Genomic and Precision Medicine

Human HCM phenotype corroborates the critical role of N-terminus of cardiac troponin I

The phenotype of *TNNI3* p.Arg21Cys-related cardiomyopathy in human carriers is consistent with prior studies on *TNNI3* p.Arg21Cys knock-in mouse models. The N-terminus of cardiac troponin I consists of a 32-amino-acid extension that is evolutionary conserved and unique to cardiac troponin I (missing from skeletal isoforms).[14] Functionally, this region has been associated directly to the binding of protein kinase (PKA) to a consensus RRRSS sequence upon β-adrenergic stimulation of the heart mainly through the phosphorylation of two serine residues at positions 23 and 24 of cardiac troponin I.[14] In the knock-in mouse with the p.Arg21Cys

mutation, the PKA-mediated phosphorylation of those residues secondary to β-adrenergic stimulation is abolished.[12] As a result, there is impaired lusitropy or relaxation of the cardiac myofilament.[15] This impaired myofilament relaxation kinetics predisposes the heart to abnormal diastolic dysfunction especially during periods of β-adrenergic stimulation such as strenuous physical activity, potentially precipitating arrhythmias.[15] In the families we describe, several young carriers of *TNNI3* p.Arg21Cys presented with story of sudden cardiac death during bouts of physical activity, consistent with this physiological mechanism described in the mouse model. While current medical treatments in HCM are exclusively geared at improving symptoms in obstructive disease, pilot studies have shown a potential role for calcium channel inhibition in improving early LV modeling in pre-clinical disease, but this area requires further research.[22]

American Heart Association.

Limitations

Circulation: Genomic and Precision Medicine

This study has several limitations. First, we only performed targeted testing for subclinical phenotype on few participants while the remainder of the clinical data were obtained as part of routine medical care. Second, because participants were recruited based on cascade screening, there was a heterogeneity in the way clinical care was delivered by multiple providers, and we are unable to consistently evaluate guideline-based clinical risk stratification for SCD in all participants. Third, a large portion of the study participants experienced sudden cardiac death prior to genotyping or even seeking medical care making it impossible to definitely exclude other causes of death, although this is unlikely. Fourth, while we investigated the occurrence of sudden cardiac death in carriers, we did not systematically evaluate the impact of the mutation on other common sequalae in HCM such as heart failure, stroke, and atrial fibrillation. Fifth, we observe significant variability in incidence of sudden cardiac death even among members of the same

family, which raises the possibility of monogenic, polygenic, or non-genetic modifiers of the phenotype.

Conclusion

TNNI3 p.Arg21Cys-related cardiomyopathy has a founder effect in south Lebanon and causes a malignant phenotype characterized by early-onset sudden cardiac death, sometimes in the absence of significant hypertrophy. Phenotypic findings in patients with the mutation corroborate the important molecular role of the N-terminus of cardiac troponin I in calcium handling and diastolic relaxation of the cardiac sarcomere. Genetically informed risk stratification and management for prevention of sudden cardiac death is challenging due to incomplete penetrance and variable expressivity, but this rare mutation is one example where this might be possible.

American Heart Association.

Acknowledgments: The authors would like to thank the patients and families for their participation in the study.

Circulation: Genomic and Precision Medicine

Sources of Funding: This study was funded by the Dubai Harvard Foundation for Medical Research (ACF, GN, FFB, JGS, CES), the National Institutes of Health (ACF, JGS, CES), the Howard Hughes Medical Institute (CES), and the Wellcome Trust [107469/Z/15/Z] (JSW).

Disclosures: ACF is a consultant and owns shares in Goodpath, which was not involved in the study. JSW reports receiving grants and personal fees from MyoKardia outside the submitted work. JGS and CES are founders and own shared in Myokardia, a company that is developing therapeutics that target the sarcomere, which was not involved in this study.

References:

1. Seidman CE, Seidman JG. Identifying sarcomere gene mutations in hypertrophic cardiomyopathy: a personal history. *Circ Res*. 2011;108:743-50.

2. Ho CY, Day SM, Ashley EA, Michels M, Pereira AC, Jacoby D, Cirino AL, Fox JC, Lakdawala NK, Ware JS, et al. Genotype and Lifetime Burden of Disease in Hypertrophic

Cardiomyopathy: Insights from the Sarcomeric Human Cardiomyopathy Registry (SHaRe). *Circulation*. 2018;138:1387-1398.

3. Weissler-Snir A, Allan K, Cunningham K, Connelly KA, Lee, DS, Spears DA, Rakowski H, Dorian P. Hypertrophic Cardiomyopathy-Related Sudden Cardiac Death in Young People in Ontario. *Circulation*. 2019; 140:1706-1716.

4. Jayaraman R, Reinier K, Nair S, Aro AL, Uy-Evanado A, Rusinaru C, Stecker EC, Gunson K, Jui J, Chugh SS. Risk Factors of Sudden Cardiac Death in the Young: Multiple-Year Community-Wide Assessment. *Circulation*. 2018;137:1561-1570.

5. Tower-Rader A, Desai MY. Phenotype-Genotype Correlation in Hypertrophic Cardiomyopathy: Less Signal, More Noise? *Circ Cardiovasc Imaging*. 2017;10.

6. Mogensen J, Murphy RT, Kubo T, Bahl A, Moon JC, Klausen IC, Elliott PM, McKenna WJ. Frequency and clinical expression of cardiac troponin I mutations in 748 consecutive families with hypertrophic cardiomyopathy. *JACC*. 2004;44:2315-25.

7. Maron MS, Rowin EJ, Wessler BS, Mooney PJ, Fatima A, Patel P, Koethe BC, Romashko M, Link MS, Maron BJ. Enhanced American College of Cardiology/American Heart Association Strategy for Prevention of Sudden Cardiac Death in High-Risk Patients With Hypertrophic Cardiomyopathy. *JAMA Cardiol*. 2019;4:644-657.

8. Gersh BJ, Maron BJ, Bonow RO, Dearani JA, Fifer MA, Link MS, Naidu SS, Nishimura RA, Ommen SR, Rakowski H, et al. 2011 ACCF/AHA guideline for the diagnosis and treatment of hypertrophic cardiomyopathy: executive summary: a report of the American College of Cardiology Foundation/American Heart Association Task Force on Practice Guidelines. *Circulation*. 2011;124:2761-96.

Circulation: Genomic and Precision Medicine

9. Murphy RT, Mogensen J, Shaw A, Kubo T, Hughes S, McKenna WJ. Novel mutation in cardiac troponin I in recessive idiopathic dilated cardiomyopathy. *Lancet*. 2004;363:371-2.

10. Mogensen J, Kubo T, Duque M, Uribe W, Shaw A, Murphy R, Gimeno JR, Elliott P, McKenna WJ. Idiopathic restrictive cardiomyopathy is part of the clinical expression of cardiac troponin I mutations. *JCI*. 2003;111:209-16.

11. Arad M, Penas-Lado M, Monserrat L, Maron BJ, Sherrid M, Ho CY, Barr S, Karim A, Olson TM, Kamisago M, et al. Gene mutations in apical hypertrophic cardiomyopathy. *Circulation*. 2005;112:2805-11.

12. Wang Y, Pinto JR, Solis RS, Dweck D, Liang J, Diaz-Perez Z, Ge Y, Walker JW, Potter JD. Generation and functional characterization of knock-in mice harboring the cardiac troponin I-R21C mutation associated with hypertrophic cardiomyopathy. *J Biol Chem*. 2012;287:2156-67.

13. Gomes AV, Harada K, Potter JD. A mutation in the N-terminus of troponin I that is associated with hypertrophic cardiomyopathy affects the Ca(2+)-sensitivity, phosphorylation kinetics and proteolytic susceptibility of troponin. *J Mol Cell Cardiol*. 2005;39:754-65.

14. Howarth JW, Meller J, Solaro RJ, Trewhella J and Rosevear PR. Phosphorylation-dependent conformational transition of the cardiac specific N-extension of troponin I in cardiac troponin. *J Mol Biol*. 2007;373:706-22.

15. Dweck D, Sanchez-Gonzalez MA, Chang AN, Dulce RA, Badger CD, Koutnik AP, Ruiz EL, Griffin B, Liang J, Kabbaj M, et al. Long term ablation of protein kinase A (PKA)-mediated cardiac troponin I phosphorylation leads to excitation-contraction uncoupling and diastolic dysfunction in a knock-in mouse model of hypertrophic cardiomyopathy. *J Biol Chem*. 2014;289:23097-111.

16. Barbour B, Salameh P. Consanguinity in Lebanon: Prevalence, Distribution and Determinants. *J Biosoc Sci*. 2009;41:505-517.

17. Valente AM, Lakdawala NK, Powell AJ, Evans SP, Cirino AL, Orav EJ, MacRae CA, Colan SD, Ho CY. Comparison of echocardiographic and cardiac magnetic resonance imaging in hypertrophic cardiomyopathy sarcomere mutation carriers without left ventricular hypertrophy. *Circ Cardiovasc Gene*. 2013;6:230-7.

18. Hiremath P, Lawler PR, Ho JE, Correia AW, Abbasi SA, Kwong RY, Jerosch-Herold M, Ho CY, Cheng S. Ultrasonic Assessment of Myocardial Microstructure in Hypertrophic Cardiomyopathy Sarcomere Mutation Carriers With and Without Left Ventricular Hypertrophy. *Circ Heart Fail*. 2016;9.

19. Vigneault DM, Yang E, Jensen PJ, Tee MW, Farhad H, Chu L, Noble JA, Day SM, Colan SD, Russell MW, et al. Left Ventricular Strain Is Abnormal in Preclinical and Overt Hypertrophic Cardiomyopathy: Cardiac MR Feature Tracking. *Radiology*. 2019;290:640-648.

20. Maron MS, Maron BJ, Harrigan C, Buros J, Gibson CM, Olivotto I, Biller L, Lesser JR, Udelson JE, Manning WJ, et al. Hypertrophic cardiomyopathy phenotype revisited after 50 years with cardiovascular magnetic resonance. *JACC*. 2009;54:220-8.

21. Ho CY, Lopez B, Coelho-Filho OR, Lakdawala NK, Cirino AL, Jarolim P, Kwong R, Gonzalez A, Colan SD, Seidman JG, et al. Myocardial fibrosis as an early manifestation of hypertrophic cardiomyopathy. *NEJM*. 2010;363:552-63.

22. Ho CY, Lakdawala NK, Cirino AL, Lipshultz SE, Sparks E, Abbasi SA, Kwong RY, Antman EM, Semsarian C, Gonzalez A, et al. Diltiazem treatment for pre-clinical hypertrophic cardiomyopathy sarcomere mutation carriers: a pilot randomized trial to modify disease expression. *JACC Heart Fail*. 2015;3:180-8.

Circulation: Genomic and Precision Medicine

Table 1. Characteristics of patients with *TNNI3* p.Arg21Cys-related cardiomyopathy

Pedigree	Subject	*TNNI3* p.Arg21Cys carrier	Sex	Age at study time (years)	Age at HCM diagnosis (years)	Age at first SCD (years)	Clinical data
DH232-A	II-1	confirmed	M	48	48	no SCD	Echo: septal hypertrophy (17mm); EKG: nonspecific changes
DH232-A	II-5	confirmed	F	50	39	40	Echo: septal hypertrophy (19mm); EKG: left ventricular hypertrophy; had primary prevention ICD implanted and received defibrillation for ventricular tachycardia at age 40
DH232-A	II-11	confirmed	F	48	45	no SCD	Echo: severe inferior wall hypertrophy; EKG: left ventricular hypertrophy
DH232-A	II-13	confirmed	M	43	35	no SCD	EKG: left ventricular hypertrophy
DH232-A	II-15	confirmed	M	33	31	no SCD	Echo: anterior wall hypertrophy (20mm); EKG: left ventricular hypertrophy; had primary prevention ICD implanted at age 33
DH232-A	III-2	confirmed	M	26	no HCM	no SCD	Echo: normal; EKG: normal
DH232-A	III-5	confirmed	F	31	no HCM	no SCD	Echo: no hypertrophy, low tissue doppler velocities; EKG: normal
DH232-A	III-6	confirmed	F	22	no HCM	no SCD	Echo: normal; EKG: normal
DH232-A	III-7	confirmed	F	21	no HCM	no SCD	Echo: normal; EKG: normal
DH232-A	III-8	confirmed	F	17	no HCM	no SCD	Echo: normal; EKG: normal
DH232-A	III-10	confirmed	M	29	26	no SCD	Echo: anterior wall and septal hypertrophy; EKG: left ventricular hypertrophy; had primary prevention ICD implanted at age 28
DH232-A	III-11	confirmed	M	23	11	no SCD	Echo: septal hypertrophy; had primary prevention ICD implanted at age 23
DH232-A	III-15	confirmed	F	12	no HCM	no SCD	Echo: no hypertrophy, low tissue doppler velocities; EKG: normal
DH232-A	III-18	confirmed	M	13	no HCM	no SCD	Echo: normal, EKG: ST wave changes, Low tissue doppler velocities
DH232-A	III-20	confirmed	M	7	no HCM	no SCD	Echo: normal; EKG: normal
DH232-A	I-1	implied	M	dead	58	no SCD	Died at the age of 58 of stroke; no previous relevant history
DH232-A	II-3	implied	F	dead	39	39	SCD at age 39; no previous relevant history
DH232-A	II-7	implied	F	dead	30	30	SCD at age 30; no previous relevant history
DH232-A	II-8	implied	F	dead	36	36	SCD at age 36; no previous relevant history
DH232-A	III-1	implied	M	28	26	no SCD	Echo: left ventricular hypertrophy
DH232-A	III-3	implied	F	25	23	no SCD	Echo: left ventricular hypertrophy
DH232-A	III-9	implied	F	dead	16	16	SCD at age 16; no previous relevant history
DH232-A	III-14	implied	M	dead	14	14	SCD at age 14; no previous relevant history
DH232-A	III-16	implied	M	dead	10	10	SCD at age 10; no previous relevant history
DH232-A	III-17	implied	M	dead	15	15	SCD at age 15; no previous relevant history
DH232-B	II-1	confirmed	F	41	41	no SCD	Echo: apical free wall hypertrophy; EKG: ST depressions
DH232-B	I-1	implied	M	dead	45	no SCD	Died at the age of 45 of stroke; no previous relevant history
DH232-B	II-2	implied	F	dead	10	10	SCD at age 10; no previous relevant history
DH232-B	II-3	implied	F	dead	21	21	SCD at age 21; no previous relevant history
DH266	I-1	confirmed	M	64	64	no SCD	Echo: apical and lateral wall hypertrophy (14.8mm)
DH266	II-3	confirmed	M	47	47	54	Echo: mid-basal hypertrophy (14mm); EKG: left ventricular hypertrophy
DH266	II-7	confirmed	M	30	30	no SCD	Echo: mid-basal hypertrophy (13mm); EKG: left ventricular hypertrophy

DH266	II-8	confirmed	M	27	27	no SCD	Echo: mid-basal (21mm) and apical (21mm) hypertrophy; EKG: left ventricular hypertrophy
DH266	II-11	confirmed	F	33	33	33	Echo: mid-basal hypertrophy (12mm); EKG: left ventricular hypertrophy; had first cardiac arrest at age 33 that was successfully resuscitated; had a second cardiac arrest at age 45 resulted in death
DH266	III-1	confirmed	M	18	18	29	Echo: mid-basal hypertrophy (17mm) and mitral valve prolapse; EKG: left ventricular hypertrophy; SCD at age 29
DH266	III-3	confirmed	M	13	13	16	Echo: mid-basal and lateral hypertrophy (17mm); EKG: left ventricular hypertrophy
DH266	II-2	implied	M	dead	27	27	SCD at age 27; no previous relevant history
DH266	II-5	implied	M	dead	23	23	SCD at age 23; no previous relevant history
DH266	II-6	implied	M	dead	25	25	SCD at age 25; no previous relevant history
DH266	II-13	implied	F	dead	22	22	SCD at age 22; no previous relevant history; cardiac autopsy report showing cardiomyocyte dysarray
DH266	III-2	implied	M	dead	13	13	SCD at age 13; no previous relevant history
DH294	II-3	confirmed	F	51	NA	no SCD	NA
DH294	II-5	confirmed	F	47	45	no SCD	Echocardiogram: LVH, IVS thickness 13mm, posterior wall thickness 13mm
DH294	III-1	confirmed	F	22	NA	no SCD	NA
DH294	I-2	implied	F	dead	40	40	SCD at age 40; no previous relevant history
DH294	II-1	implied	F	dead	42	42	SCD at age 42; no previous relevant history
DH294	II-2	implied	F	dead	23	23	SCD at age 23; no previous relevant history
DH294	III-3	implied	M	dead	17	17	SCD at age 17; no previous relevant history
HK	II-2	confirmed	F	51	no HCM	no SCD	Echo: mild mitral regurgitation, no hypertrophy
HK	II-4	confirmed	F	49	49	no SCD	Echo: apical hypertrophy; heart failure
HK	III-1	confirmed	F	18	18	18	Echo: septal hypertrophy; SCD at age of 18
HK	III-2	confirmed	M	16	16	21	Echo: left ventricular hypertrophy; had primary prevention ICD implanted; SCD at age 21
HK	I-1	implied	M	dead	54	54	SCD at age 54; no previous relevant history
HK	II-1	implied	F	dead	45	45	SCD at age 45; no previous relevant history
HK	II-7	implied	M	dead	18	18	SCD at age 18; no previous relevant history
HK	II-8	implied	M	dead	21	21	SCD at age 21; no previous relevant history
HK	III-3	implied	F	dead	18	18	SCD at age 18; no previous relevant history

Circulation: Genomic and Precision Medicine

Figures Legends:

Figure 1. Pedigrees of families with HCM due to *TNNI3* p.Arg21Cys. DH232-A and DH232-B are related; II-1 in DH232A is first degree cousin of I-1 in DH232-B. +/- denotes presence of heterozygous *TNNI3* p.Arg21Cys mutation. Circles denote female subjects, and boxes male subjects. Black indicates subjects affected with HCM on echocardiography or subjects who suffered from Sudden Cardiac Death (SCD), white indicates normal subjects, and grey indicates that the status of the subject is unknown. A slash through the symbol denotes a deceased subject.

Figure 2. Survival of subjects with *TNNI3* p.Arg21Cys-related cardiomyopathy compared to carriers of *MYBPC3* p.Arg502Trp. Age at sudden cardiac death (SCD) for 40 subjects with *TNNI3* p.Arg21Cys-related cardiomyopathy as compared to 47 HCM patients with the *MYBPC3* p.Arg502Trp mutation shows a median age of SCD is 22.5 years in the *TNNI* p.Arg21Cys group. At that age, only about 10% of the *MYBC* p.Arg502Trp carriers have SCD.

American Heart Association.
Circulation: Genomic and Precision Medicine

Figure 3. Advanced cardiac imaging of genotype positive but phenotype negative patient. The 13-year old male (patient III-18 from DH232-A) is asymptomatic and carries the p.Arg21Cys mutation. Cardiac imaging reveals absence of hypertrophy on echocardiography (A), doppler tissue myocardial velocities at the lateral aspect of the mitral annulus (B) show reduced Ea and S velocities, suggesting early systolic and diastolic myocardial dysfunction. Cardiac MRI images in end-diastole (C-E) show a relatively asymmetrical wall thickening of the basal and mid antero-septal and infero-septal walls as compared to the lateral wall (10-11 mm vs 6-7 mm). The wall thickness per se is within normal and the LV mass total is also normal. There is uniform

nulling of the myocardium on delayed imaging post Gadolinium, including the area of maximal thickening. No evidence of scar or fibrosis or infiltrative disease were seen.

Figure 4. Advanced cardiac imaging of genotype and phenotype positive patient. The 48-year old male (patient II-1 from DH232-A) is symptomatic, carries the p.Arg21Cys mutation and has known left ventricular hypertrophy on echocardiography (arrow) (A). Doppler tissue myocardial velocities at the lateral aspect of the mitral annulus (B) show reduced Ea and S velocities, suggesting early systolic and diastolic myocardial dysfunction. Cardiac MRI (C, D) show septal hypertrophy (13-14 mm) vs. 7 mm at the lateral wall and mild to moderate focal enhancement in the mid anteroseptum consistent with scarring

American Heart Association.

Circulation: Genomic and Precision Medicine

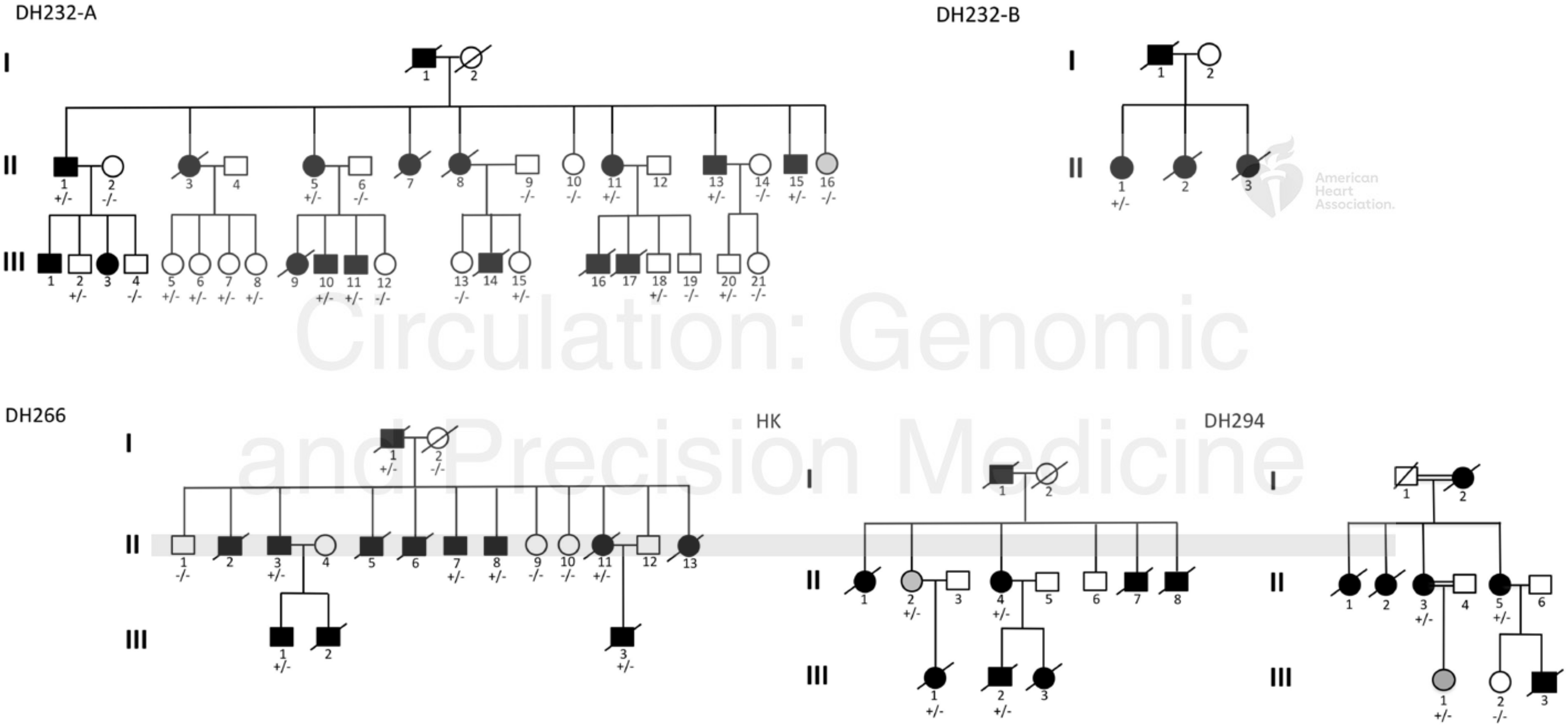

Circulation: Genomic and Precision Medicine
American Heart Association.

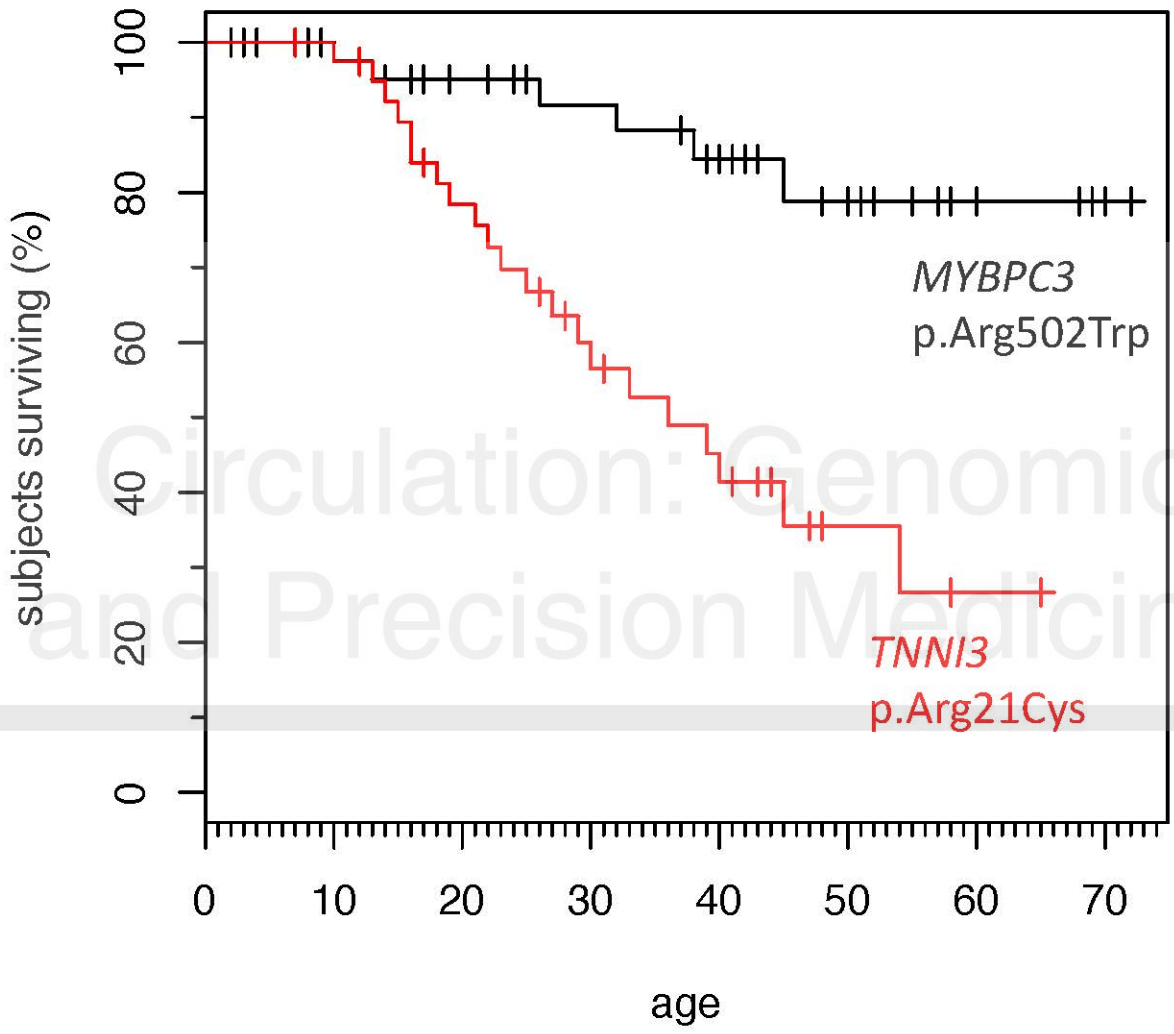

Circulation: Genomic and Precision Medicine
American Heart Association.

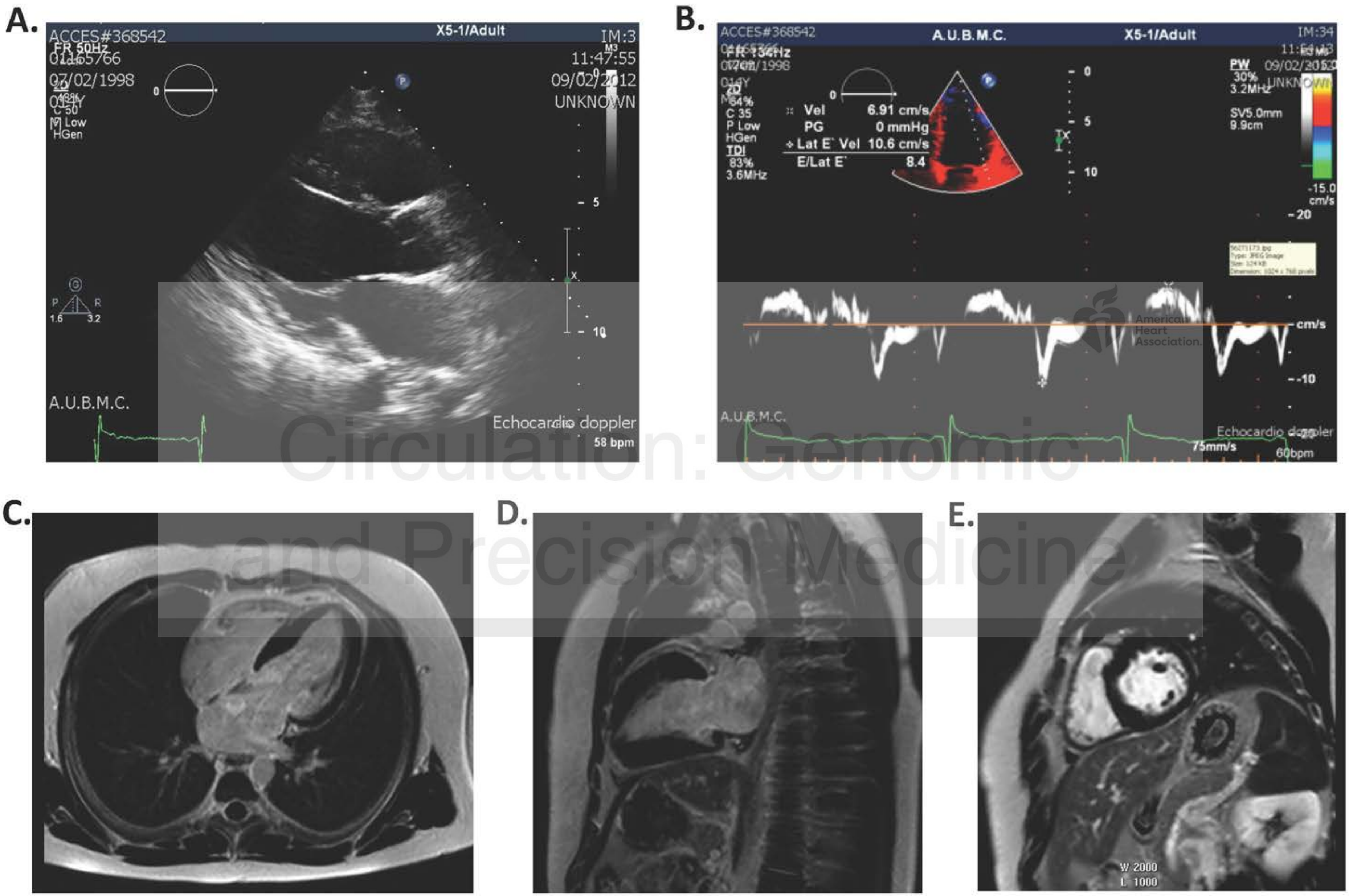

Circulation: Genomic and Precision Medicine
American Heart Association.

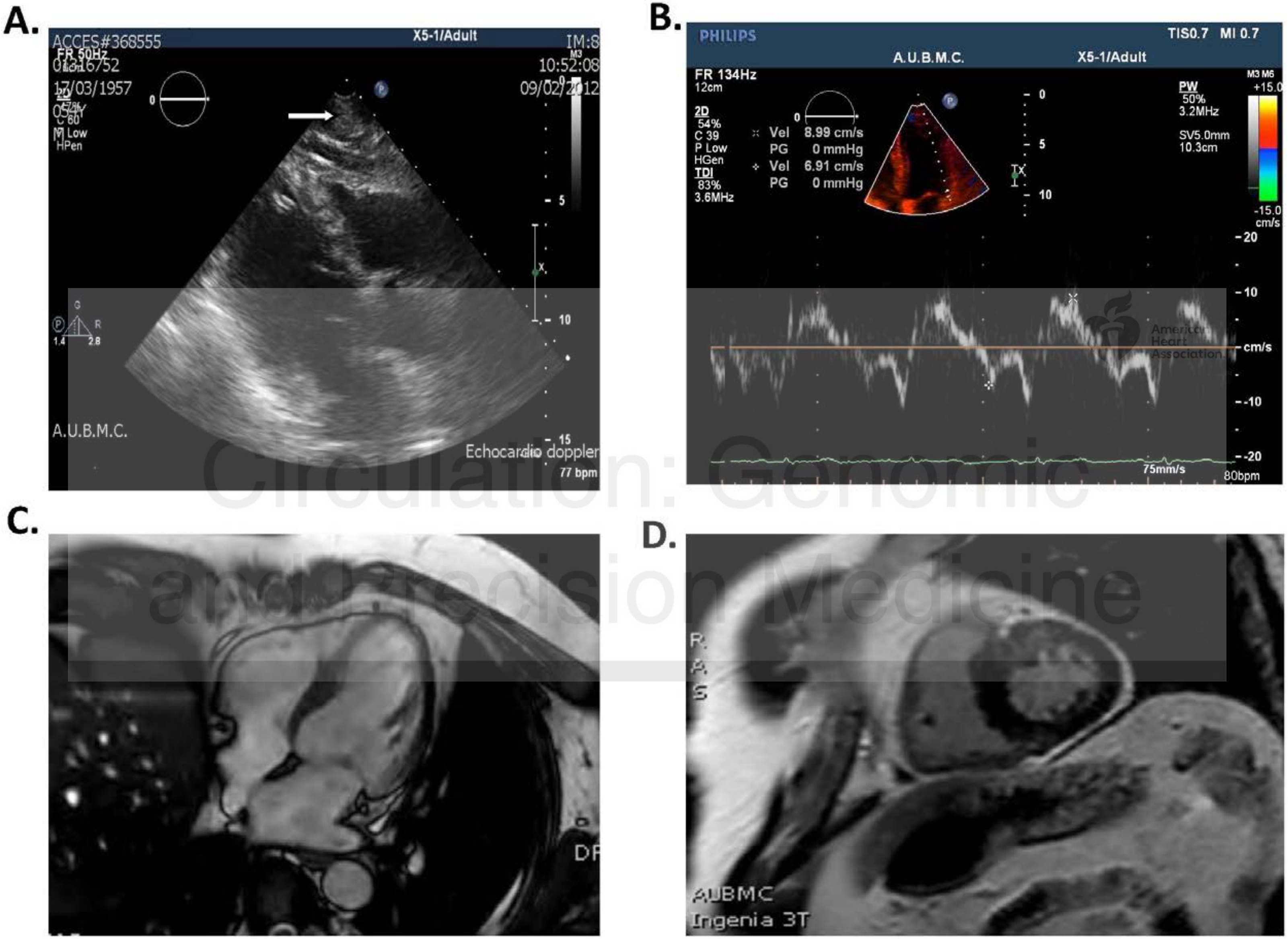

Circulation: Genomic and Precision Medicine
American Heart Association
